# Effect of Tetramethylpyrazine on Atherosclerosis and SCAP/SREBP-1c Signaling Pathway in ApoE^−/−^ Mice Fed with a High-Fat Diet

**DOI:** 10.1155/2017/3121989

**Published:** 2017-04-12

**Authors:** Ying Zhang, Pan Ren, Qunfu Kang, Weihong Liu, Sinai Li, Ping Li, Hongxu Liu, Juju Shang, Lei Zhang, Yanbing Gong, Mingxue Zhou

**Affiliations:** ^1^Department of TCM, Beijing Luhe Hospital, Capital Medical University, Beijing 100149, China; ^2^Beijing Hospital of Traditional Chinese Medicine, Capital Medical University, Beijing Institute of Traditional Chinese Medicine, Beijing 100010, China; ^3^Beijing Hospital of Traditional Chinese Medicine Shunyi Branch, Beijing 101300, China; ^4^Beijing Hospital of Traditional Chinese Medicine, Capital Medical University, Beijing 100010, China; ^5^Dongfang Hospital, Beijing University of Chinese Medicine, Beijing 100078, China

## Abstract

Lipid metabolism dysregulation plays a crucial role in the occurrence of atherosclerosis (As). SCAP/SREBP signaling is the main pathway for regulating lipid metabolism. Tetramethylpyrazine (TMP), a Traditional Chinese Medicine (TCM) for treating angina pectoris, has antiatherosclerotic effects and ameliorates blood lipids disturbance. However, its precise mechanism remains unclear. This study investigated the mechanism of TMP in ameliorating As in mice model. After six weeks of high-fat diet, 30 ApoE^−/−^ mice were randomized (*n* = 10) and treated with Lipitor, TMP, or distilled water for six weeks. The serum blood lipids and insulin levels were measured. The expressions of PAQR3, Insig-1, SCAP, SREBP-1c, IRS-1, PI3K, Akt, and mTORC-1 in the adipose tissues were determined. The results showed that TMP could significantly decrease blood lipids levels, insulin, and corrected plaque area of the ApoE^−/−^ mice as compared to the untreated mice (*P* < 0.05, *P* < 0.01). Moreover, TMP could significantly downregulate the expressions of SCAP, SREBP-1c, PAQR3, IRS-1, PI3K, Akt, and mTORC1 (*P* < 0.01). Thus, TMP may ameliorate lipid metabolism disorder and As by downregulating PAQR3 and inhibiting SCAP/SREBP-1c signaling pathway. In addition, PI3K/Akt/mTORC1 signaling pathway may be involved in this process.

## 1. Introduction

Atherosclerosis (As) is the leading cause of death worldwide in cardiovascular diseases, and it is also a primary contributor to the pathogenesis of acute cardiovascular diseases including myocardial infarctions [1, 2]. Although important advances in the understanding of the molecular mechanisms of As have been made in the last few decades [3], many questions remain. Therefore, it is crucial to investigate the mechanism of As formation for the treatment of As-related arterial diseases. Dysregulation of lipid homeostasis is closely related to the pathogenesis of As and has become a major underlying reason for its development [4]. Recent studies reported that SCAP/sterol regulatory element-binding proteins (SREBP) complex is crucial to control lipid homeostasis [5], and SREBPs, as key transcriptional factors, can regulate the biosynthesis of cholesterol [6]. Insig, as an accepted anchor protein of SCAP/SREBP complex, is capable of retaining SCAP/SREBP in the endoplasmic reticulum (ER). In addition, recent study reported that progestin and adipoQ receptors 3 (PAQR3), as a novel anchoring protein of SCAP/SREBP complex, also can regulate cholesterol biosynthesis and SREBP activation [7].

Insulin resistance plays a crucial role in the As process. Insulin induces forceful effects on lipid accumulation and regulates the expressions of genes involved in lipogenesis [8]. The stimulatory effect induced by insulin on lipogenesis was significantly inhibited after treatment with PI3K/Akt/mTORC1 inhibitors [8]. Moreover, PI3K/Akt/mTORC1 signaling conversely exerts crucial effects on the maintaining integrity of lipid rafts by modulating SREBP activation and subsequent cholesterogenesis [6].

Tetramethylpyrazine (TMP), as an active ingredient from Traditional Chinese Medicine (TCM), has been used for the treatment of As for a long time. TMP has been reported to be effective for the treatment of ischemia-reperfusion injury and ischemic brain injury [9, 10]. TMP contributes to expanding blood vessels, increasing coronary and cerebral blood flow, preventing platelet aggregation, and improving microcirculation [11]. In addition, TMP exerts a tangible protective effect in vascular endothelial cells and might be a potential protective agent for As [3]. However, the mechanism of TMP in As and lipid metabolism remains unknown. Based on the important role of PAQR3 and SCAP/SREBP signaling pathway in lipid metabolism, we investigated the effect of TMP on PAQR3 and SCAP/SREBP signaling pathway in order to explore its antiatherosclerotic mechanisms.

## 2. Materials and Methods

### 2.1. Animals

The ApoE^−/−^ mice (*n* = 30, 8 weeks of age, male, weighing 18–20 g) with C57BL/6J background were introduced and bred by the Department of Laboratory Animal Science of Peking University Health Science Center. All animal research conformed to the Guide for the Care and Use of Laboratory Animals published by the US National Institutes of Health (NIH Publication number 85-23) and was approved by the Ethics Review Board for Animal Studies of Peking University Health Science Center (Permit number IMM-GuYC-1).

### 2.2. Reagents

The blood lipid kits were purchased from Zhongsheng Beikong Biotechnology Co., Ltd. (Beijing, China), to measure total cholesterol (TC), triglycerides (TG), LDL, and high-density lipoprotein (HDL-C). A TRIzol kit was purchased from Invitrogen (California), polymerase chain reaction (PCR) primers were obtained from Sangon Biotech Co., Ltd. (Shanghai, China), and an M-MLV RT kit and a real-time- (RT-) PCR kit was purchased from Takara Company (Otsu, Shiga, Japan).

### 2.3. Establishment of the As Model

Thirty ApoE^−/−^ mice were fed with a high-fat diet containing 21% (wt/wt) fat from lard supplemented with 0.15% (wt/wt) cholesterol obtained from Beijing Ke'aoXieli Feed Co. Ltd. (Beijing, China) for 12 weeks [12].

### 2.4. Drug Treatment

After six weeks on a high-fat diet, the ApoE^−/−^ mice were randomized (10 per group) and treated with Lipitor (positive control drug, 3 mg/kg), TMP (45.05 mg/kg), or distilled water (control group) by intragastric administration, for an additional six weeks accompanied by a high-fat diet. The choice of drug doses was based on the clinically relevant doses in humans (the conversion coefficient between humans and mice is 9.01; the doses in mice are 9.01 times the clinical doses in humans) [13]. Distilled water was used to dilute the drugs.

### 2.5. Histology

After six weeks of drug therapy, all mice were euthanized by intraperitoneal injection with 0.1% pentobarbital sodium. The hearts were extracted, and one-third of the heart was fixed in 10% formaldehyde to determine the morphology of any atherosclerotic plaque by hematoxylin and eosin (HE) staining. The aorta, liver, and abdominal adipose tissues of the mice were removed and stored at −80°C [14, 15].

### 2.6. Determination of Lipid Concentration

After six weeks of drug therapy, all mice were euthanized by intraperitoneal injection with 0.1% pentobarbital sodium. Blood samples were drawn from the left ventricle of all ApoE^−/−^ mice that had received a high-fat diet for 12 weeks. The serum TC and TG levels were determined. The LDL and HDL were determined by immunoturbidimetry. Finally, all indices were determined using the RX-2000 radiometer (Technicon Instruments Company, Tarrytown, New York) [14, 15].

### 2.7. Real-Time PCR

After six weeks of drug therapy, all mice were euthanized by intraperitoneal injection with 0.1% pentobarbital sodium. The total RNA from the aorta, liver, and adipose tissue was extracted using a TRIzol kit according to the manufacturer's instructions. The primers for PAQR3, Insig-1, SCAP, SREBP1, and GAPDH are shown in Table 1. The protocol for RT-PCR was previously described by us [16].

### 2.8. Western Blotting

After six weeks of drug therapy, all mice were euthanized by intraperitoneal injection with 0.1% pentobarbital sodium. The adipose tissue from the other three groups of mice were removed and stored at −80°C to examine the protein expressions of Insig-1, SCAP, and SREBP-1c. The protocol for western blotting was previously described by us [17]. Primary antibodies including Insig-1 (Abcam, ab70784, 1 : 200), SREBP-1c (Abcam, ab28481, 1 : 1000), SCAP (Abcam, ab19013, 1 : 1000), IRS-1 (Abcam, ab52167, 1 : 1000), PI3K (Abcam, ab151549, 1 : 1000), Akt (Abcam, ab8805, 1 : 500), mTORC1(Abcam, ab2732, 1 : 200), PAQR3 (Abcam, ab174327, 1 : 250), and *β*-actin (Beijing Zhongshan Golden Bridge Biotechnology Co., TA-09, 1 : 1000) were used in this study. Protein expression was detected with an enhanced chemiluminescence detection system (Vigorous, Beijing, China).

### 2.9. Statistical Analysis

Mean values and standard deviations were calculated for each tested variable. All statistical analyses were performed using SPSS 11.5. Normally distributed data were analyzed using one-way analysis of variance with a Bonferroni post hoc test to evaluate the statistical significance of intergroup differences in all tested variables. In all cases, statistical significance was set at *P* < 0.05 [14, 15].

## 3. Results

### 3.1. Feeding Fat Provokes the Formation of Atherosclerotic Plaques

**Figure 1:**
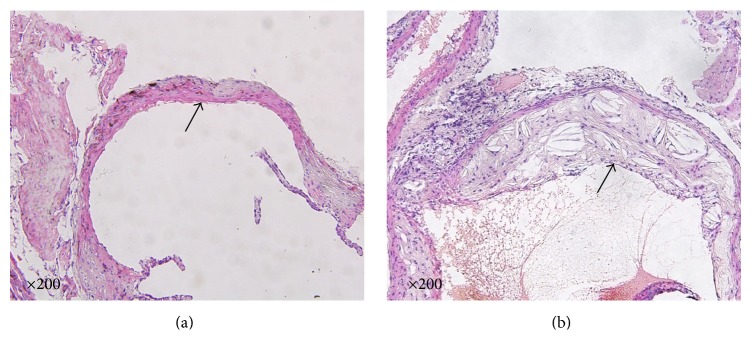
Comparison of pathological morphology of the aorta of C57BL/6J mice and ApoE^−/−^ mice 13 weeks after being fed with different diets. (a) Hematoxylin and eosin (HE) showing the pathological morphology of the aorta of C57BL/6J mice fed with a regular diet. (b) The pathological morphology of the aorta of ApoE^−/−^ mice fed with a high-fat diet. The black arrow indicates the aorta of the mice. Scale bar = 200 *μ*m.

After the ApoE^−/−^ mice were fed with a high-fat diet for 12 weeks, atherosclerotic plaques in the aortic valves attachment sites, including cholesterol crystals and foam cells, were observed in the aortic roots, whereas no plaques were observed in the aortic roots of the C57 mice (Figure 1).

### 3.2. TMP Ameliorates Blood Lipids and Atherosclerotic Plaques

**Figure 2:**
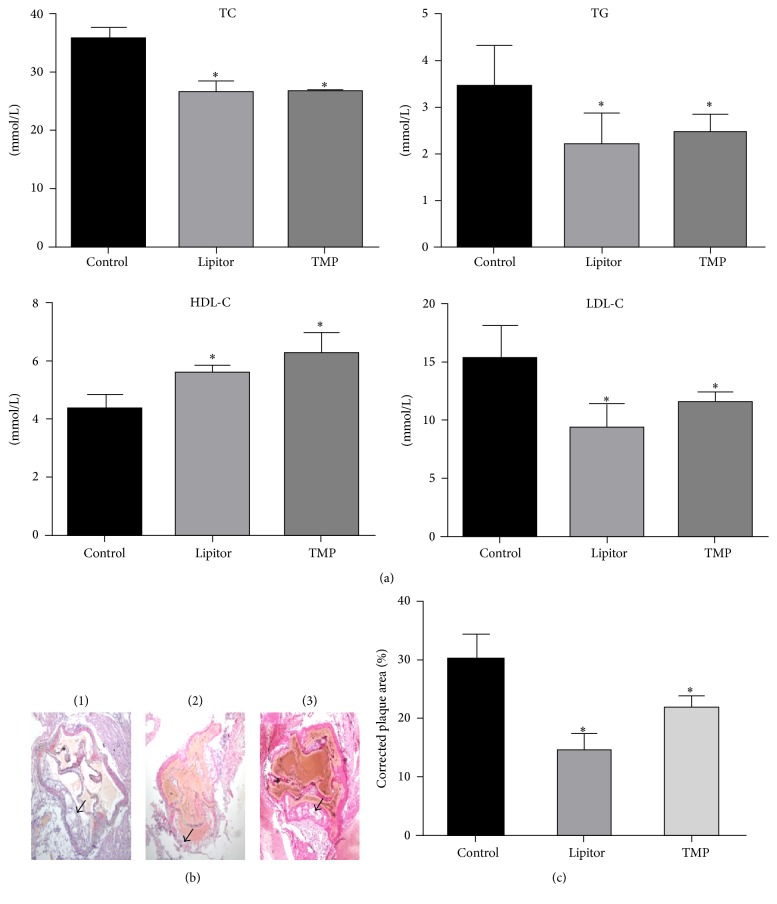
Tetramethylpyrazine (TMP) ameliorates blood lipids and atherosclerotic plaques. (a) TMP ameliorates the serum lipids disturbance in ApoE^−/−^ mice fed with a high-fat diet. TC, total cholesterol; TG, triglycerides; HDL-C, high-density lipoprotein cholesterol; LDL-C, low-density lipoprotein cholesterol. ^*∗*^*P* < 0.05 versus the control group, *n* = 10. (b) Hematoxylin and eosin (HE) staining showing the pathological changes in the atherosclerotic plaque in the aorta of ApoE^−/−^ mice after treatment with TMP. (1) ApoE^−/−^ group; (2) ApoE^−/−^ + Lipitor group; (3) ApoE^−/−^ + TMP group. Scale bars = 200 *μ*m; the black arrow indicates the atherosclerotic plaque in aorta. (c) Statistical analysis of the corrected area of atherosclerotic plaque of ApoE^−/−^ mice after the TMP treatment. ^*∗*^*P* < 0.01 versus the control group; *n* = 10.

As shown in Figure 2(a), the serum levels of TG, TC, and LDL-C in the TMP and Lipitor groups were significantly decreased (*P* < 0.05), and the serum levels of HDL-C in the TMP and Lipitor groups were significantly increased as compared to the control group. There was no significant difference between the two drug-treatment groups (*P* > 0.05). HE staining showed that the corrected areas of atherosclerotic plaques in the TMP and Lipitor groups were significantly decreased as compared to the control group (*P* < 0.01), with no significant difference between the two drug-treatment groups (*P* > 0.05) (Figures 2(b) and 2(c)).

### 3.3. TMP Reduces Weight and Insulin Level

**Figure 3:**
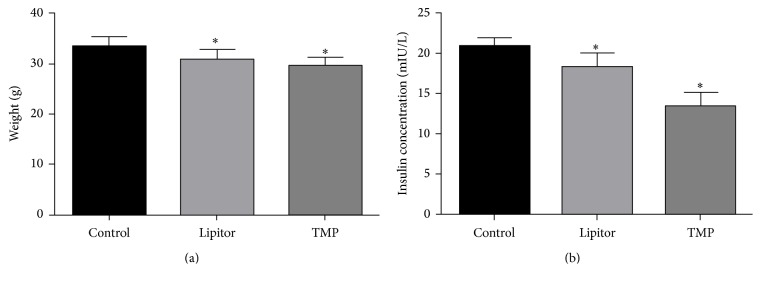
TMP reduces weight and insulin level. (a) Statistical analysis of weight of ApoE^−/−^ mice after TMP treatment. (b) Statistical analysis of insulin levels of ApoE^−/−^ mice after TMP treatment. ^*∗*^*P* < 0.05 versus the control group; *n* = 10.

The weight and serum insulin levels of the TMP and Lipitor group mice were significantly decreased as compared to the control group (*P* < 0.05), with no significant difference between the two drug-treatment groups (*P* > 0.05) (Figure 3).

### 3.4. TMP Downregulates mRNA Expressions of Insig, PAQR3, SCAP, and SREBP1 in the Aorta, Liver, and Adipose Tissues

**Figure 4:**
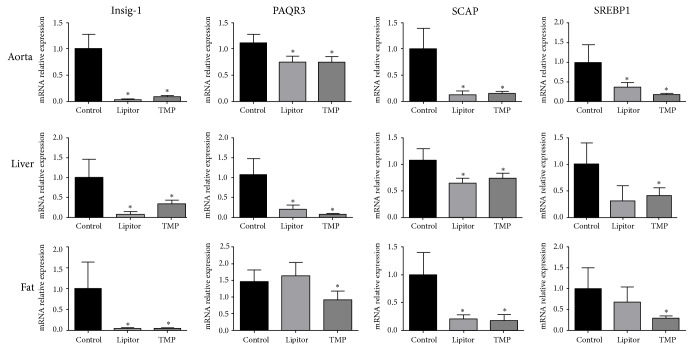
TMP downregulates the mRNA expressions of Insig-1, PAQR3, SCAP, and SREBP-1 in the aorta, liver, and adipose tissues. ^*∗*^*P* < 0.01 versus the control group; *n* = 4.

Six weeks after the drug treatment of the ApoE^−/−^ mice fed with a high-fat diet, the mRNA expressions of Insig, PAQR3, SCAP, and SREBP in the aorta, liver, and adipose tissue of the TMP group mice were significantly decreased as compared to the control group (*P* < 0.01), with no significant difference between the two drug-treatment groups (*P* > 0.05) (Figure 4).

### 3.5. TMP Downregulates the Protein Expressions of Insig-1, PAQR3, SREBP-1c, and SCAP

**Figure 5:**
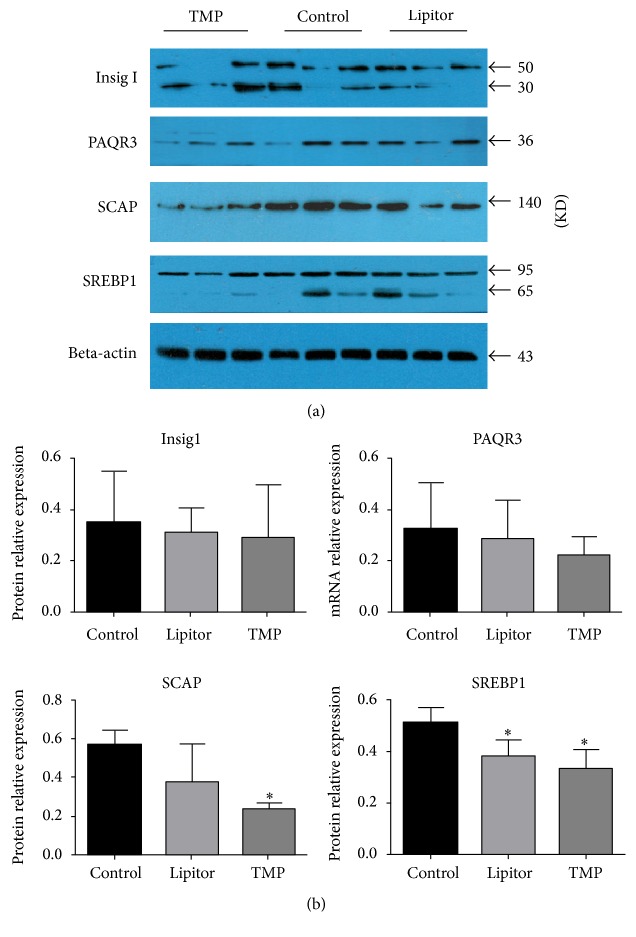
TMP downregulates the protein expressions of Insig-1, PAQR3, SREBP-1c, and SCAP in adipose tissues. (a) Western blotting shows the protein expressions of Insig-1, PAQR3, SREBP1-c, and SCAP in adipose tissues of ApoE^−/−^ mice fed with a high-fat diet. (b) Statistical analysis graph showing the protein expressions of Insig-1, PAQR3, SREBP-1c, and SCAP in the adipose tissues. ^*∗*^*P* < 0.05 versus the control group; *n* = 3.

Six weeks after the drug treatment of the ApoE^−/−^ mice fed with a high-fat diet, the protein expressions of SREBP1-c and SCAP in the adipose tissue of the TMP group mice were significantly decreased (*P* < 0.05). The protein expressions of Insig-1 and PAQR3 in the adipose tissue of the TMP group mice were slightly reduced (*P* > 0.05). There was no significant difference between the two drug-treatment groups (*P* > 0.05) (Figure 5).

### 3.6. TMP Downregulates the Protein Expressions of IRS-1, PI3K, p-Akt, and mTORC1 in Adipose Tissues

**Figure 6:**
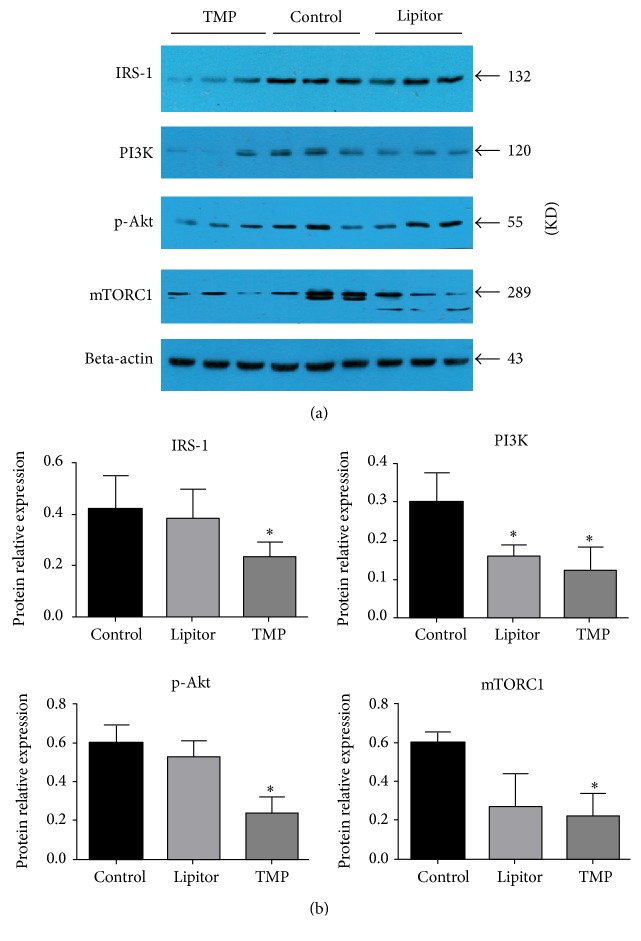
TMP downregulates the protein expressions of IRS-1, PI3K, p-Akt, and mTORC1 in the adipose tissues. (a) Western blotting shows the protein expressions of IRS-1, PI3K, p-Akt, and mTORC1 in adipose tissues of ApoE^−/−^ mice fed with a high-fat diet. (b) Statistical analysis graph showing the protein expressions of IRS-1, PI3K, p-Akt, and mTORC1 in adipose tissues. ^*∗*^*P* < 0.05 versus the control group; *n* = 3.

Six weeks after TMP treatment of the ApoE^−/−^ mice fed with a high-fat diet, the protein expressions of IRS-1, PI3K, p-Akt, and mTORC1 in the adipose tissues of TMP group mice were significantly decreased as compared to the control group (*P* < 0.05) (Figure 6).

## 4. Discussion 

This study demonstrated that TMP can dramatically ameliorate As in ApoE^−/−^ mice fed with a high-fat diet by attenuating the blood lipid levels and reducing the plaque areas. In addition, TMP can inhibit the progress of As and ameliorate lipid metabolism disorder by downregulating PAQR3 and inhibiting SCAP/SREBP-1c signaling pathway in these mice. Moreover, PI3K/Akt/mTORC1 signaling pathway may be involved in this process.

In TCM, Chuan Xiong (*Ligusticum wallichii* Franchat) is applied for the treatment of cardiovascular diseases. TMP, a purified and chemically identified component of Chuan Xiong, has been reported to be effective for the treatment of kidney and brain damage induced by ischemia-reperfusion injury (I/R) [9, 10, 18]. This study showed that TMP can reduce the atherosclerotic plaques and attenuate abnormal levels of blood lipids in ApoE^−/−^ mice fed with a high-fat diet, which is consistent with a previous report [11].

Among the mechanisms associated with As formation, the disturbance of lipid metabolism is widely accepted. Liver and adipose tissue are mainly involved in the process of lipid metabolism. Cholesterol biosynthesis can be regulated by SREBP and its escort protein SCAP [7]. SREBPs are pivotal activators of key enzymes responsible for the biosynthesis of fatty acids and cholesterol and play a crucial role in the progress of As [19, 20]. Under optimal cholesterol conditions, Insigs act as anchor proteins to retain SCAP/SREBP in the ER [7]. This study showed that TMP can downregulate the mRNA and protein expressions of SCAP and SREBP in the aorta, liver, and adipose tissue of ApoE^−/−^ mice fed with a high-fat diet. This suggests that TMP may exert its antiatherosclerotic effect and ameliorate the disturbance of lipid metabolism by inhibiting SCAP/SREBP signaling pathway. In addition, TMP also inhibits the expression of Insig-1 in the adipose tissue of ApoE^−/−^ mice fed with a high-fat diet. PAQR3 is a member of the progestin and adipoQ receptors (PAQR) superfamily [21]. A recent study showed that PAQR3 can interact with SCAP and SREBP and promote SCAP/SREBP complex formation, potentiate SREBP processing, and further enhance lipid synthesis [7]. Another study also showed that after feeding with a high-fat diet, the levels of blood cholesterol and LDL-C in PAQR3-deleted mice were significantly reduced as compared to the wild-type mice [22]. Thus, PAQR3, as an anchor protein for SCAP/SREBP complex, plays a vital role in the regulation of cholesterol homeostasis and SREBP pathway. This study showed that TMP can downregulate the mRNA expression of PAQR3 and partially reduce its protein expression in the adipose tissue of ApoE^−/−^ mice fed with a high-fat diet. This indicates that PAQR3, and not Insig-1, may be a crucial target of TMP for ameliorating the disturbance of lipid metabolism. In addition, TMP may exert its antiatherosclerotic effect by downregulating PAQR3 and further inhibiting SCAP/SREBP-1c signaling pathway.

Insulin resistance plays a crucial role in the process of As. It has been confirmed that high-fat diet can induce insulin resistance in various mouse strains [23–26]. Insulin has been confirmed to induce forceful effects on lipid accumulation and the expression of genes involved in lipogenesis [7]. In this study, the weight and serum levels of insulin in the TMP and Lipitor groups were significantly decreased as compared to the control group. This suggests that TMP may ameliorate insulin resistance in ApoE^−/−^ mice fed with a high-fat diet.

PI3K/Akt/mTOR signaling pathway is the pivotal pathway for regulating insulin resistance. The stimulatory effect of insulin on lipogenesis has been significantly inhibited after treating with PI3K/Akt/mTOR inhibitors [7]. PI3K/Akt/mTORC1 signaling pathway can maintain the integrity of lipid rafts by regulating SREBP activation [5]. In addition, PAQR3 can also modulate insulin sensitivity in mice partly via negative regulation of PI3K [22, 27]. This study showed that TMP can downregulate the protein expressions of IRS-1, PI3K, p-Akt, and mTORC1 in adipose tissues. This suggests that induction of PI3K/Akt/mTORC1 signaling pathway may be involved in the TMP-mediated amelioration of the lipid metabolism disturbance by inhibiting SCAP/SREBP signaling pathway.

In conclusion, we showed that TMP could inhibit the progress of As and ameliorate lipid metabolism disorder by downregulating PAQR3 and inhibiting SCAP/SREBP-1c signaling pathway in ApoE^−/−^ mice fed with a high-fat diet. In addition, PI3K/Akt/mTORC1 signaling pathway may be involved in this process.

## Figures and Tables

**Table 1:** The primers in this study.

Gene	Forward	Reverse
Insig	5′-GGAGAGGACTCTTCCACAGC-3′	5′-GTCCAACGCACATAGGACAC-3′
Paqr3	5′-GATGGCATTGGATTATGCAG-3′	5′-AAGCACGGTGATCAGGTACA-3′
Scap	5′-GAAGTCATCGGTGTCTCCCT-3′	5′-GCTGTCTCTCAGCACATGGT-3′
Srebp	5′-TTGTGGAGCTCAAAGACCTG-3′	5′-TGCAAGAAGCGGATGTAGTC-3′
